# Supplementary material for: Multi‐dataset identification of innovative feature genes and molecular mechanisms in keratoconus
Source: J Cell Mol Med. 2024 Sep 19;28(18):e70079. doi: 10.1111/jcmm.70079 (PMC11412914; doi:10.1111/jcmm.70079)
Supplement: Supplementary file 2 — Material S2. [file JCMM-28-e70079-s002.zip › 7.GSEA analysis/4.MYRF/KEGG_REGULATION_OF_ACTIN_CYTOSKELETON.html]

Details for gene set KEGG\_REGULATION\_OF\_ACTIN\_CYTOSKELETON[GSEA]

|  || Dataset | MYRF.group.cls#h\_versus\_l |
| Phenotype | group.cls#h\_versus\_l |
| Upregulated in class | l |
| GeneSet | KEGG\_REGULATION\_OF\_ACTIN\_CYTOSKELETON |
| Enrichment Score (ES) | -0.33484033 |
| Normalized Enrichment Score (NES) | -1.4348524 |
| Nominal p-value | 0.026156941 |
| FDR q-value | 0.21578649 |
| FWER p-Value | 0.842 |
Table: GSEA Results Summary

  

Fig 1: Enrichment plot: KEGG\_REGULATION\_OF\_ACTIN\_CYTOSKELETON      
 Profile of the Running ES Score & Positions of GeneSet Members on the Rank Ordered List

  

| SYMBOL | TITLE | RANK IN GENE LIST | RANK METRIC SCORE | RUNNING ES | CORE ENRICHMENT || 1 | PIK3R5 | na | 661 | 0.528 | -0.0006 | No |
| 2 | INSRR | na | 1368 | 0.412 | -0.0066 | No |
| 3 | PDGFRA | na | 1428 | 0.407 | 0.0060 | No |
| 4 | ITGA4 | na | 1618 | 0.389 | 0.0141 | No |
| 5 | RAC3 | na | 2468 | 0.325 | 0.0009 | No |
| 6 | ITGAL | na | 2764 | 0.308 | 0.0032 | No |
| 7 | FGF8 | na | 2858 | 0.303 | 0.0111 | No |
| 8 | BDKRB2 | na | 2872 | 0.302 | 0.0213 | No |
| 9 | CYFIP2 | na | 3408 | 0.276 | 0.0155 | No |
| 10 | ARHGEF6 | na | 3595 | 0.268 | 0.0195 | No |
| 11 | CHRM3 | na | 4941 | 0.224 | -0.0116 | No |
| 12 | FGF18 | na | 5499 | 0.207 | -0.0205 | No |
| 13 | FGF16 | na | 5776 | 0.199 | -0.0215 | No |
| 14 | ITGA9 | na | 5848 | 0.197 | -0.0167 | No |
| 15 | SOS1 | na | 6706 | 0.172 | -0.0354 | No |
| 16 | FN1 | na | 6837 | 0.168 | -0.0333 | No |
| 17 | PIK3R1 | na | 7105 | 0.161 | -0.0354 | No |
| 18 | FGF5 | na | 7739 | 0.145 | -0.0487 | No |
| 19 | APC2 | na | 8076 | 0.137 | -0.0536 | No |
| 20 | FGF3 | na | 8177 | 0.134 | -0.0518 | No |
| 21 | BRAF | na | 8198 | 0.133 | -0.0477 | No |
| 22 | TMSB4Y | na | 8430 | 0.129 | -0.0499 | No |
| 23 | ROCK1 | na | 8751 | 0.121 | -0.0549 | No |
| 24 | FGFR4 | na | 8772 | 0.121 | -0.0512 | No |
| 25 | FGF12 | na | 8823 | 0.120 | -0.0485 | No |
| 26 | ARPC4 | na | 8885 | 0.119 | -0.0461 | No |
| 27 | GSN | na | 9023 | 0.115 | -0.0460 | No |
| 28 | BAIAP2 | na | 9266 | 0.110 | -0.0492 | No |
| 29 | ITGB7 | na | 9321 | 0.109 | -0.0469 | No |
| 30 | KRAS | na | 9371 | 0.108 | -0.0445 | No |
| 31 | CHRM5 | na | 9436 | 0.107 | -0.0426 | No |
| 32 | DIAPH2 | na | 9565 | 0.104 | -0.0427 | No |
| 33 | BCAR1 | na | 9571 | 0.104 | -0.0392 | No |
| 34 | PFN4 | na | 9614 | 0.103 | -0.0368 | No |
| 35 | F2 | na | 9622 | 0.103 | -0.0334 | No |
| 36 | PIK3CG | na | 9794 | 0.100 | -0.0348 | No |
| 37 | ITGAD | na | 9801 | 0.099 | -0.0315 | No |
| 38 | PIKFYVE | na | 9889 | 0.098 | -0.0306 | No |
| 39 | FGF17 | na | 10236 | 0.092 | -0.0374 | No |
| 40 | FGF13 | na | 10299 | 0.091 | -0.0360 | No |
| 41 | MYH14 | na | 10451 | 0.087 | -0.0373 | No |
| 42 | MYL5 | na | 10477 | 0.087 | -0.0350 | No |
| 43 | LIMK1 | na | 10531 | 0.086 | -0.0335 | No |
| 44 | TMSB4X | na | 11192 | 0.075 | -0.0500 | No |
| 45 | PIK3CA | na | 11255 | 0.074 | -0.0492 | No |
| 46 | VAV2 | na | 11454 | 0.071 | -0.0525 | No |
| 47 | VAV3 | na | 11728 | 0.067 | -0.0580 | No |
| 48 | PIP4K2B | na | 11849 | 0.066 | -0.0592 | No |
| 49 | ITGB6 | na | 12239 | 0.060 | -0.0684 | No |
| 50 | CD14 | na | 12315 | 0.059 | -0.0685 | No |
| 51 | ACTB | na | 12485 | 0.057 | -0.0714 | No |
| 52 | PIK3R2 | na | 12535 | 0.056 | -0.0708 | No |
| 53 | IQGAP1 | na | 12574 | 0.056 | -0.0700 | No |
| 54 | SOS2 | na | 12783 | 0.053 | -0.0741 | No |
| 55 | FGF20 | na | 12795 | 0.053 | -0.0726 | No |
| 56 | ARHGAP35 | na | 13305 | 0.046 | -0.0857 | No |
| 57 | SSH3 | na | 13440 | 0.045 | -0.0881 | No |
| 58 | ARHGEF4 | na | 13623 | 0.042 | -0.0919 | No |
| 59 | CSK | na | 13727 | 0.041 | -0.0934 | No |
| 60 | ROCK2 | na | 13790 | 0.040 | -0.0938 | No |
| 61 | FGF9 | na | 13888 | 0.039 | -0.0952 | No |
| 62 | PDGFA | na | 13993 | 0.038 | -0.0969 | No |
| 63 | MAP2K2 | na | 14012 | 0.038 | -0.0961 | No |
| 64 | ARPC5 | na | 14172 | 0.036 | -0.0994 | No |
| 65 | MYLK | na | 14280 | 0.035 | -0.1013 | No |
| 66 | MAPK3 | na | 14382 | 0.034 | -0.1031 | No |
| 67 | FGFR3 | na | 14422 | 0.033 | -0.1030 | No |
| 68 | IQGAP3 | na | 14494 | 0.033 | -0.1039 | No |
| 69 | IQGAP2 | na | 14572 | 0.032 | -0.1051 | No |
| 70 | DIAPH3 | na | 14601 | 0.032 | -0.1048 | No |
| 71 | WAS | na | 14632 | 0.031 | -0.1045 | No |
| 72 | DIAPH1 | na | 14672 | 0.031 | -0.1046 | No |
| 73 | ACTN4 | na | 14881 | 0.029 | -0.1096 | No |
| 74 | ARPC3 | na | 15089 | 0.027 | -0.1146 | No |
| 75 | FGF7 | na | 15518 | 0.023 | -0.1262 | No |
| 76 | ABI2 | na | 15640 | 0.021 | -0.1290 | No |
| 77 | ARPC5L | na | 15651 | 0.021 | -0.1285 | No |
| 78 | CHRM2 | na | 15691 | 0.021 | -0.1289 | No |
| 79 | TIAM1 | na | 15716 | 0.021 | -0.1289 | No |
| 80 | MYL12B | na | 15972 | 0.018 | -0.1357 | No |
| 81 | F2R | na | 16254 | 0.015 | -0.1433 | No |
| 82 | SSH2 | na | 16648 | 0.012 | -0.1543 | No |
| 83 | EGFR | na | 17031 | 0.008 | -0.1650 | No |
| 84 | PIK3CB | na | 17199 | 0.007 | -0.1696 | No |
| 85 | RAC2 | na | 17259 | 0.006 | -0.1711 | No |
| 86 | MYH10 | na | 17326 | 0.006 | -0.1728 | No |
| 87 | WASL | na | 17383 | 0.005 | -0.1743 | No |
| 88 | TIAM2 | na | 17643 | 0.003 | -0.1817 | No |
| 89 | NCKAP1 | na | 17696 | 0.002 | -0.1831 | No |
| 90 | NCKAP1L | na | 17704 | 0.002 | -0.1832 | No |
| 91 | ARPC2 | na | 17815 | 0.001 | -0.1864 | No |
| 92 | PAK4 | na | 17893 | 0.001 | -0.1886 | No |
| 93 | MYL12A | na | 18052 | -0.001 | -0.1931 | No |
| 94 | ARPC1A | na | 18115 | -0.002 | -0.1949 | No |
| 95 | PPP1CB | na | 18335 | -0.004 | -0.2011 | No |
| 96 | PPP1CA | na | 18340 | -0.004 | -0.2010 | No |
| 97 | WASF2 | na | 18382 | -0.004 | -0.2021 | No |
| 98 | FGF19 | na | 18545 | -0.006 | -0.2066 | No |
| 99 | FGF10 | na | 18767 | -0.008 | -0.2127 | No |
| 100 | RHOA | na | 18827 | -0.008 | -0.2141 | No |
| 101 | EGF | na | 19142 | -0.012 | -0.2228 | No |
| 102 | CFL1 | na | 19157 | -0.012 | -0.2228 | No |
| 103 | ITGB4 | na | 19208 | -0.013 | -0.2238 | No |
| 104 | PAK3 | na | 19225 | -0.013 | -0.2238 | No |
| 105 | FGF11 | na | 19615 | -0.017 | -0.2344 | No |
| 106 | CYFIP1 | na | 19792 | -0.019 | -0.2389 | No |
| 107 | MOS | na | 20086 | -0.023 | -0.2465 | No |
| 108 | GIT1 | na | 20104 | -0.023 | -0.2462 | No |
| 109 | MYH9 | na | 20365 | -0.026 | -0.2528 | No |
| 110 | MAPK1 | na | 20406 | -0.027 | -0.2531 | No |
| 111 | PIK3CD | na | 20433 | -0.027 | -0.2529 | No |
| 112 | PIK3R3 | na | 20464 | -0.027 | -0.2528 | No |
| 113 | DOCK1 | na | 20532 | -0.028 | -0.2538 | No |
| 114 | FGF14 | na | 20664 | -0.030 | -0.2565 | No |
| 115 | FGFR2 | na | 20983 | -0.033 | -0.2646 | No |
| 116 | ENAH | na | 21068 | -0.034 | -0.2658 | No |
| 117 | PIP5K1C | na | 21353 | -0.038 | -0.2727 | No |
| 118 | PPP1R12A | na | 21522 | -0.040 | -0.2762 | No |
| 119 | RAF1 | na | 21720 | -0.042 | -0.2804 | No |
| 120 | ARHGEF12 | na | 21776 | -0.043 | -0.2805 | No |
| 121 | BRK1 | na | 21804 | -0.043 | -0.2798 | No |
| 122 | PAK6 | na | 21811 | -0.043 | -0.2784 | No |
| 123 | ITGB2 | na | 21824 | -0.044 | -0.2772 | No |
| 124 | HRAS | na | 21856 | -0.044 | -0.2766 | No |
| 125 | RAC1 | na | 21936 | -0.045 | -0.2773 | No |
| 126 | ACTG1 | na | 22317 | -0.050 | -0.2866 | No |
| 127 | PFN3 | na | 22341 | -0.050 | -0.2855 | No |
| 128 | PFN1 | na | 22403 | -0.051 | -0.2855 | No |
| 129 | PPP1CC | na | 22860 | -0.057 | -0.2967 | No |
| 130 | WASF1 | na | 23182 | -0.062 | -0.3038 | No |
| 131 | CDC42 | na | 23297 | -0.064 | -0.3048 | No |
| 132 | PTK2 | na | 23650 | -0.070 | -0.3126 | No |
| 133 | ITGA3 | na | 23712 | -0.071 | -0.3118 | No |
| 134 | ITGB5 | na | 23730 | -0.071 | -0.3098 | No |
| 135 | VCL | na | 24000 | -0.076 | -0.3150 | No |
| 136 | PAK2 | na | 24412 | -0.082 | -0.3240 | No |
| 137 | FGF22 | na | 24497 | -0.084 | -0.3235 | No |
| 138 | LIMK2 | na | 24514 | -0.084 | -0.3210 | No |
| 139 | PIP4K2A | na | 24991 | -0.093 | -0.3315 | Yes |
| 140 | GNA12 | na | 25107 | -0.095 | -0.3315 | Yes |
| 141 | PFN2 | na | 25127 | -0.095 | -0.3287 | Yes |
| 142 | ITGA2 | na | 25150 | -0.096 | -0.3260 | Yes |
| 143 | FGD1 | na | 25157 | -0.096 | -0.3228 | Yes |
| 144 | GNG12 | na | 25293 | -0.099 | -0.3233 | Yes |
| 145 | CRK | na | 25398 | -0.101 | -0.3227 | Yes |
| 146 | SCIN | na | 25628 | -0.105 | -0.3257 | Yes |
| 147 | FGD3 | na | 25733 | -0.107 | -0.3249 | Yes |
| 148 | ITGA2B | na | 25913 | -0.111 | -0.3262 | Yes |
| 149 | RDX | na | 26199 | -0.117 | -0.3304 | Yes |
| 150 | FGF21 | na | 26242 | -0.118 | -0.3275 | Yes |
| 151 | PAK1 | na | 26325 | -0.120 | -0.3256 | Yes |
| 152 | RRAS2 | na | 26466 | -0.122 | -0.3254 | Yes |
| 153 | PDGFB | na | 26504 | -0.123 | -0.3222 | Yes |
| 154 | PIP4K2C | na | 26803 | -0.130 | -0.3262 | Yes |
| 155 | ITGAV | na | 26862 | -0.132 | -0.3233 | Yes |
| 156 | FGF6 | na | 27010 | -0.135 | -0.3228 | Yes |
| 157 | GNA13 | na | 27072 | -0.137 | -0.3198 | Yes |
| 158 | ARPC1B | na | 27124 | -0.138 | -0.3164 | Yes |
| 159 | PDGFRB | na | 27235 | -0.141 | -0.3146 | Yes |
| 160 | RRAS | na | 27376 | -0.144 | -0.3136 | Yes |
| 161 | EZR | na | 27423 | -0.146 | -0.3099 | Yes |
| 162 | ARHGEF7 | na | 27516 | -0.148 | -0.3073 | Yes |
| 163 | FGF2 | na | 27758 | -0.154 | -0.3089 | Yes |
| 164 | FGF1 | na | 27813 | -0.156 | -0.3050 | Yes |
| 165 | ACTN3 | na | 27890 | -0.157 | -0.3017 | Yes |
| 166 | ACTN1 | na | 27985 | -0.160 | -0.2988 | Yes |
| 167 | ITGA11 | na | 28180 | -0.166 | -0.2986 | Yes |
| 168 | MYLK3 | na | 28223 | -0.167 | -0.2940 | Yes |
| 169 | PXN | na | 28410 | -0.173 | -0.2933 | Yes |
| 170 | ARAF | na | 28444 | -0.174 | -0.2882 | Yes |
| 171 | MSN | na | 28445 | -0.174 | -0.2821 | Yes |
| 172 | MYL9 | na | 28537 | -0.176 | -0.2785 | Yes |
| 173 | MYLK2 | na | 28598 | -0.178 | -0.2740 | Yes |
| 174 | ITGAE | na | 28615 | -0.178 | -0.2683 | Yes |
| 175 | APC | na | 28648 | -0.180 | -0.2629 | Yes |
| 176 | VAV1 | na | 28682 | -0.181 | -0.2575 | Yes |
| 177 | CRKL | na | 29092 | -0.194 | -0.2626 | Yes |
| 178 | ITGA7 | na | 29295 | -0.200 | -0.2614 | Yes |
| 179 | ITGA8 | na | 29346 | -0.201 | -0.2558 | Yes |
| 180 | ITGA6 | na | 29414 | -0.203 | -0.2506 | Yes |
| 181 | SLC9A1 | na | 29528 | -0.207 | -0.2466 | Yes |
| 182 | ITGB3 | na | 29529 | -0.207 | -0.2394 | Yes |
| 183 | ITGB1 | na | 29873 | -0.219 | -0.2416 | Yes |
| 184 | NRAS | na | 30019 | -0.224 | -0.2380 | Yes |
| 185 | FGF23 | na | 30092 | -0.227 | -0.2321 | Yes |
| 186 | ACTN2 | na | 30149 | -0.229 | -0.2257 | Yes |
| 187 | MAP2K1 | na | 30150 | -0.229 | -0.2177 | Yes |
| 188 | INS | na | 30231 | -0.232 | -0.2119 | Yes |
| 189 | MYL2 | na | 30601 | -0.246 | -0.2140 | Yes |
| 190 | BDKRB1 | na | 30628 | -0.248 | -0.2060 | Yes |
| 191 | CHRM1 | na | 30926 | -0.260 | -0.2055 | Yes |
| 192 | ITGB8 | na | 30931 | -0.260 | -0.1965 | Yes |
| 193 | ARHGEF1 | na | 30936 | -0.260 | -0.1875 | Yes |
| 194 | SSH1 | na | 31117 | -0.270 | -0.1833 | Yes |
| 195 | MYL7 | na | 31601 | -0.295 | -0.1869 | Yes |
| 196 | ITGAM | na | 31711 | -0.302 | -0.1795 | Yes |
| 197 | ITGA10 | na | 32096 | -0.323 | -0.1793 | Yes |
| 198 | PDGFD | na | 32544 | -0.354 | -0.1798 | Yes |
| 199 | MYL10 | na | 33204 | -0.407 | -0.1846 | Yes |
| 200 | CFL2 | na | 33257 | -0.414 | -0.1716 | Yes |
| 201 | ITGAX | na | 33560 | -0.452 | -0.1646 | Yes |
| 202 | PIP5K1A | na | 33731 | -0.475 | -0.1528 | Yes |
| 203 | ITGA1 | na | 33768 | -0.482 | -0.1370 | Yes |
| 204 | PIP5K1B | na | 33965 | -0.515 | -0.1246 | Yes |
| 205 | CHRM4 | na | 34199 | -0.571 | -0.1113 | Yes |
| 206 | FGF4 | na | 34351 | -0.616 | -0.0941 | Yes |
| 207 | MRAS | na | 34392 | -0.631 | -0.0731 | Yes |
| 208 | ITGA5 | na | 34505 | -0.679 | -0.0526 | Yes |
| 209 | PDGFC | na | 34611 | -0.758 | -0.0290 | Yes |
| 210 | FGFR1 | na | 34693 | -0.924 | 0.0010 | Yes |
Table: GSEA details [plain text format]

  

Fig 2: KEGG\_REGULATION\_OF\_ACTIN\_CYTOSKELETON      
 Blue-Pink O' Gram in the Space of the Analyzed GeneSet

  

Fig 3: KEGG\_REGULATION\_OF\_ACTIN\_CYTOSKELETON: Random ES distribution      
 Gene set null distribution of ES for **KEGG\_REGULATION\_OF\_ACTIN\_CYTOSKELETON**
